# Measuring development assistance for health systems strengthening and health security: an analysis using the Creditor Reporting System database

**DOI:** 10.12688/f1000research.24012.1

**Published:** 2020-06-09

**Authors:** Jessica Kraus, Gavin Yamey, Marco Schäferhoff, Hugo Petitjean, Jessica Hale, Kenan Karakulah, Chris Kardish, Estuardo Pineda, Francesca Sanders, Naomi Beyeler, Sara Fewer, Rachel Nugent, Dean T. Jamison, Ben Oppenheim, Indermit Gill

**Affiliations:** 1 SEEK Development, Berlin, Germany; 2 Duke Global Health Institute, Duke University, Durham, North Carolina, USA; 3 Open Consultants, Berlin, Germany; 4 RTI International, Research Triangle Park, North Carolina, USA; 5 Duke Center for International Development, Duke Univesity, Durham, North Carolina, USA; 6 adelphi, Berlin, Germany; 7 Global Health Group, University of California, San Francisco, San Francesco, California, USA; 8 Institute of Global Health Sciences, University of California, San Francisco, San Francisco, California, USA; 9 Metabiota, San Francisco, California, USA

**Keywords:** Development assistance, development assistance for health, health systems strengthening, health security, pandemic preparedness, universal health coverage, DAH, UHC

## Abstract

**Background**: Health systems strengthening (HSS) and health security are two pillars of universal health coverage (UHC). Investments in these areas are essential for meeting the Sustainable Development Goals and are of heightened relevance given the emergence of the 2019 novel coronavirus disease (COVID-19). This study aims to generate information on development assistance for health (DAH) for these areas, including how to track it and how funding levels align with country needs.

**Methods**: We developed a framework to analyze the amount of DAH disbursed in 2015 for the six building blocks of the health system (‘system-wide HSS’) plus health security (emergency preparedness, risk management, and response) at both the global (transnational) and country level. We reviewed 2,427 of 32,801 DAH activities in the Creditor Reporting System (CRS) database (80% of the total value of disbursements in 2015) and additional public information sources. Additional aid activities were identified through a keyword search.

**Results**: In 2015, we estimated that US$3.1 billion (13.4%) of the US$22.9 billion of DAH captured in the CRS database was for system-wide HSS and health security: US$2.5 billion (10.9%) for system-wide HSS, mostly for infrastructure, and US$0.6 billion (2.5%) for system-wide health security. US$567.1 million (2.4%) was invested in supporting these activities at the global level. If responses to individual health emergencies are included, 7.5% of total DAH (US$1.7B) was for health security. We found a correlation between DAH for HSS and maternal mortality rates, and we interpret this as evidence that HSS aid generally flowed to countries with greater need.

**Conclusions** **:** Achieving UHC by 2030 will require greater investments in system-wide HSS and proactive health emergency preparedness. It may be appropriate for donors to more prominently consider country needs and global functions when investing in health security and HSS.

## Introduction

In September 2015, United Nations member states adopted the Sustainable Development Goals (SDGs), including SDG3 – to “ensure healthy lives and promote well-being for all at all ages” ^1^. Within SDG3 is the ambitious target of achieving universal health coverage (UHC) by 2030 (SDG target 3.8). Defined by the World Health Organization (WHO) as “all individuals and communities receive the health services they need without suffering financial hardship” ^2^.

A core foundation of achieving target 3.8 is health systems strengthening (HSS) ^3^, which Kutzin and Sparkes argue “comprises the means” by which the objectives of UHC can be achieved ^4^. A related foundation is health security – emergency preparedness, risk management, and response capabilities ^5– 7^. The incremental investments needed for HSS and health security are substantial. Estimates exist on the resources available for funding certain core components of UHC (such as health systems and pandemic preparedness). Stenberg and colleagues estimate that by 2030 the additional annual funding needed to reach the health-related SDGs across 67 low-income and middle-income countries would be US$274-371 billion, of which three quarters is needed for HSS (and one quarter for program support) ^8^. A more recent estimate from the Disease Control Priorities Project found incremental costs to be about the same (although slightly lower) than did Sternberg and colleagues but that specific program investments account for a much larger fraction of the total (57% rather than 25%) with correspondingly smaller needs for generalized HSS. The DCP estimated the cost for pandemic preparedness to be about US$4 billion per year out of its total estimated cost ^9^. The Commission on a Global Health Risk Framework for the Future estimates that an additional US$3.4 billion annually is needed “to upgrade national pandemic preparedness capabilities” ^10^.

There are several debates on how development assistance for health (DAH) can best be used to support the health SDGs. Essential to informing these debates is knowing how donor support is currently spent. There has been little research on how much DAH flows to HSS and health security. The Institute for Health Metrics and Evaluation (IHME) publishes annual estimates of global health financing flows, including by year, focus area, donor, and channel, as well as projections on future spending ^11^. IHME found that in 2015, 9.4% of total DAH went towards HSS and 0.8% was directed at pandemic preparedness ^12^. However, given its methodology and scope, IHME’s financing global health estimate cannot be disaggregated to the activity level nor heavily filtered (e.g., to estimate DAH for specific building blocks of the health system, for system-wide health security support, etc.) IHME researchers occasionally provide one-off analyses on specific sub-area(s), e.g., on donor financing for human resources for health ^13^. Little research has also been done on how estimates compare to country need.

This study provides evidence on donor support for system-wide HSS and system-wide health security, two key UHC pillars. To the best of our knowledge, this study is the first to assess flows of DAH towards these two foundations of UHC in a way that allows the findings to be disaggregated by HSS building block. By providing an in-depth analysis of DAH in 2015, the first year of the SDGs, our study can serve as a baseline for monitoring global progress. In addition, to facilitate evidence-based decision-making, we compared flows against proxies for country needs. By reviewing activities manually through a line-by-line review, financing can be scrutinized and classified in greater detail. Basing the study on a sample of the publicly available, activity level financing data reported to the Organisation for Economic Cooperation and Development’s Creditor Reporting System (CRS) strengthens transparency. Through this approach, we have made available a complementary dataset that we hope can facilitate both donors and recipient countries to better understand which specific activities are contributing to UHC through system-wide HSS and health security.

Our study is designed to inform the global health discourse. There is a continued discussion of the conditions under which DAH for program-specific intervention support (a ‘vertical’ approach), program strengthening (a ‘diagonal’ approach), or system-wide investments (a ‘horizontal’ approach) is more effective ^14^. There is also continued discussion in the global health community on the value of DAH for “global functions” (activities that have transnational benefits, e.g., pandemic preparedness) versus DAH given to a single country for disease control. The *Lancet* Commission on Investing in Health and the WHO’s Common Goods for Health program suggest that DAH should be increasingly directed to global functions ^15^. These analyses suggest that global functions remain seriously underfunded and that country-specific aid for routine functions can lead countries to relocate domestic resources away from health (‘fungibility’). To inform these discussions, we developed a framework that disaggregates between global versus country-specific functions and distinguishes between system-wide investments and other approaches to health support.

## Methods

**Figure 1.**
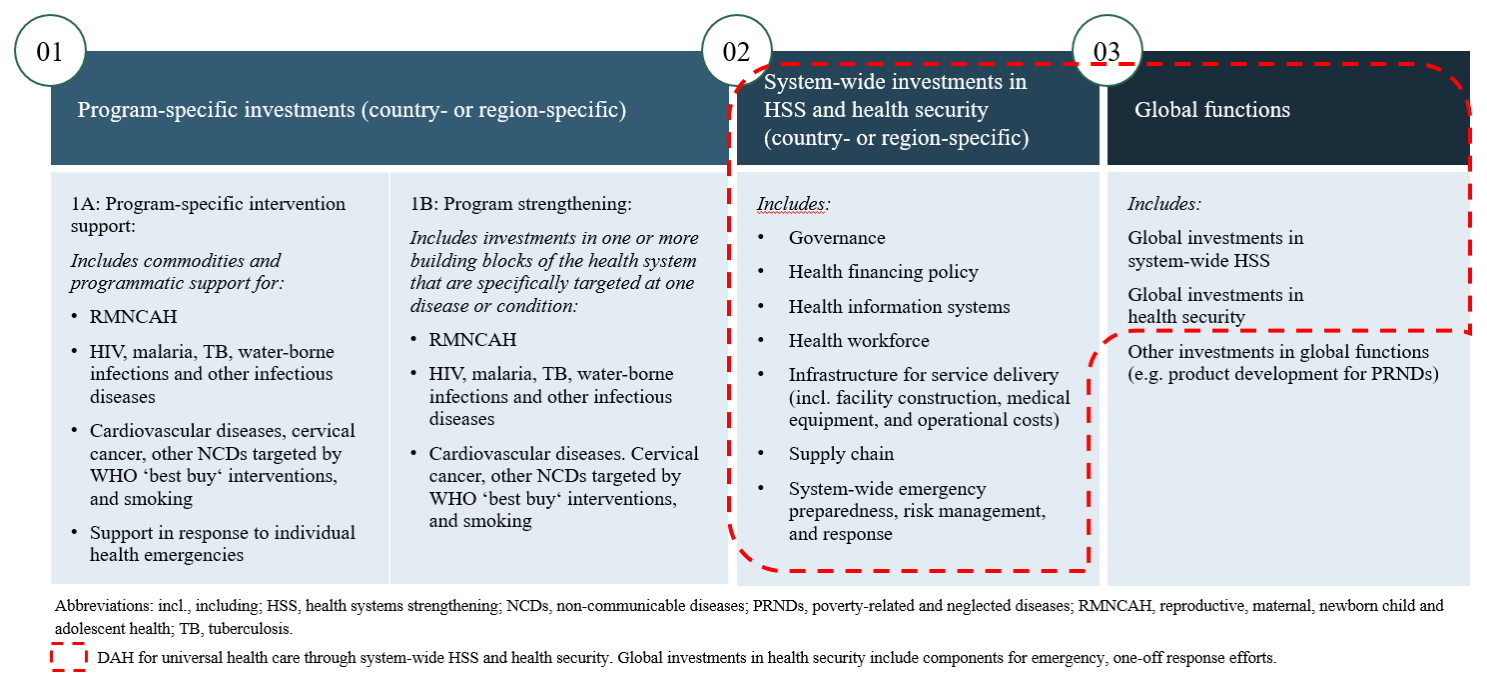
Analytic framework for classification of development assistance for health (DAH) into three health investment areas.

We developed a new approach for tracking DAH for HSS and health security. We began by developing an analytic framework that classifies DAH aid activity across three health investment areas ( Figure 1). The first is country-level program-specific investments (Box 1 of Figure 1), which focus on a single disease/response program, such as DAH given to a country to establish an HIV drug supply system. The second is country-level, system-wide HSS and health security, i.e., investments with a focus across diseases, beyond a single disease response effort (Box 2 of Figure 1). An example is an investment in a comprehensive national health information system. The third is investments in ‘global functions’ – DAH that has transnational benefits (Box 3 of Figure 1), e.g., a platform to foster information exchange across a region ^16– 18^.

Each of these three categories was broken down into sub-categories. Program-specific investments were broken down into either program-specific intervention support (Box 1A of Figure 1) or program strengthening (Box 1B of Figure 1). Program-specific intervention support refers to targeted programmatic support (e.g., commodities for a specific disease program). Program-specific investments were further broken down across four sub-categories: three disease areas (reproductive, maternal, newborn, child, and adolescent health [RMNCAH]; infectious diseases; and non-communicable diseases [NCDs]) and a fourth category, programmatic support for one-off health emergencies (e.g., equipment for an Ebola outbreak). Program strengthening investments (Box 1B of Figure 1) aim to improve the quality of a disease-specific program by strengthening just one or a few building blocks of the health system (i.e., diagonal funding). Program strengthening investments were also divided into RMNCAH, infectious diseases, or NCDs.

Program-specific investments contrast with system-wide HSS (Box 2 of Figure 1), which strengthens the health system in ways that improve health outcomes across multiple diseases and conditions. System-wide HSS investments were broken out across the six building blocks of the health system presented in Stenberg *et al.*’s conceptual framework ^8^. We also included a component for ‘system-wide emergency preparedness, risk management and response’ for activities that strengthened capacity to deal with epidemic and pandemic preparedness and response, e.g., a mobile emergency alert system.

Finally, global functions (Box 3 of Figure 1) comprised three sub-categories of investments with transnational benefits: HSS (e.g., knowledge exchange on HSS), health security (e.g., research and development of medical countermeasures to control pandemics), and ‘other investments in global functions’ (e.g., in product development for neglected diseases). Appendix, Tables 1–4 (see *Extended data*) ^19^, provide further detail on the sub-categories.

To estimate total DAH for UHC through system-wide HSS and health security, we summed the investments for the sub-components highlighted in Figure 1.

### Applying the analytic framework to the Creditor Reporting System database

We conducted a detailed analysis of aid activities from the Creditor Reporting System (CRS) database, complemented by a review of additional information sources, to estimate DAH flows to health security and HSS in the year 2015, the latest year for which data were available at the time of the analysis. The CRS is a publicly available database that provides information about DAH resource flows. It covers spending from official bilateral and multilateral organizations – including official development assistance (ODA) from Development Assistance Committee (DAC) members and some non-members – as well as from private philanthropic foundations (at the time of the analysis, limited to the Bill & Melinda Gates Foundation). Unlike other sources, the CRS database is scrutinized by the Organisation for Economic Cooperation and Development (OECD). It avoids double-counting and provides information on individual aid activities (Appendix p. 18, see *Extended data*) ^19, 20^.

We used our analytic framework to code aid activities in the CRS database. We used the OECD’s June 2017 update of the 2015 CRS database (see *Source data)*, the most recent year at the time of the analysis. We covered ODA, as well as grant financing provided by the Bill & Melinda Gates Foundation. When donors report to the CRS, they assign projects to codes ^21^. DAH was defined using the DAC’s sector codes for health, general (code 121), basic health (122), and population policies/programmes and reproductive health (130) ^22^, as well as social mitigation of HIV (16064). These sectors have previously been used to define health aid ^23, 24^. All financing levels were calculated using the ‘USD_Disbursement’ column (provided in US$ 2015 constant prices). At the time of our analysis, donors reported US$22.9 billion in development assistance for these codes in 2015. Each row in this dataset represents one aid activity. The sample of aid activities was selected by ordering the 32,801 "aid activities" from highest to lowest in dollar terms using the ‘USD Disbursement’ column, and taking those that represent 80% of all health ODA. This resulted in a sample of 2,427 aid activities (USD$18.3 billion, or 80% of total health ODA). A non-probability sampling method was chosen to capture the majority of DAH. Our approach to extrapolation and reviewing aid activities was based on our earlier effort to track donor financing for health by function ^12^.

A team of analysts assessed what proportion of the aid activity went to each of the investment areas. Measures were put in place to ensure accuracy and consistency across the team of analysts. We developed a codebook with definitions and examples for each area in our analytical framework. Health aid activities were reviewed based on the descriptions in the CRS. Descriptions in a language other than English or French were translated through Google Translate. When descriptive information in the CRS was insufficient to adequately code a project, we obtained additional project information from publicly available sources, e.g., budgets and project logframes from the websites of donors and project implementers. The best source of additional information was typically a donor’s website. When no information was provided on a donor or a recipient’s website, other sources of information were sought, e.g., news articles. Based on the information found, the aid activity amount was then broken down by sub-category.

Analysts noted the sources and rationale for each aid activity’s allocations. In the best-case scenario, a coder was able to find specific financial information, e.g. budget details of the disbursement or a detailed aid activity document outlining where funding was going. For example, if an aid activity document showed that $300,000 was spent on health information systems of an aid activity worth $1,500,000, then 20% was allocated to health information systems and this would be entered as a formula (“=300,000/1,500,000”). For cases in which no financial data for specific aid activities was identified, the classification was based on non-financial information: calculations were made proportionately to the number of activities presented in each aid activity’s descriptive information, with each objective weighted equally. For example, if no budget information could be identified and five objectives were located, three that relate to emergency preparedness, one that relates to health information systems, and another that relates to global investments in HSS, then 60% was entered in the health security column, 20% to health information systems, and 20% to global investments in HSS.

However, given that entries in the CRS database oftentimes are multi-dimensional in nature and do not include enough information for an in-depth analysis, a field was provided for analysts to indicate how certain they were of the aid activity’s classification. A project manager was available to check uncertain classifications against the codebook. In cases of discrepancy, the activity’s classification was discussed and agreed upon as a team.

Given the contributions that other sectors make to HSS and health security (e.g., support for humanitarian emergencies and water supply improvements), to arrive at a high-level estimate of total donor support for HSS and health security, we expanded our analysis and conducted a light-touch analysis of additional CRS purpose codes) ^25^. We conducted a key term search using Python 3.6 across 14 additional CRS purpose codes: 14030; 14031; 14032; 14050; 15110; 16010; 16050; 16062; 52010; 72010; 72040; 72050; 73010; 74010 ^26^. The following keywords were used to identify relevant projects: Accountability System; Antimicrobial Resistan; Care Service; Cholera; Clinic; Clinical Service; Cold Chain; Conditional Cash Transfer; Continuum of Care; Coronavirus; Crimean Congo; Detection; Diagnos-; Diagnostic; Disease; Drug; Early Warning; Ebola; Emergency Management; Epidemic; Essential Intervention; Facil-; Fever; Financial Management System; Financial Risk Protection; Haemorrhagic Fever; Health; Hendra; Hospital; HSS; Hygiene; Illness; Infectio-; Influenza; Information System; Inventory; Laborator-; Lassa; Leptospirosis; Malnutrition; Marburg; Meningitis; MERS; Morbidity; Mortality; Nipah; Outbreak; Pandemic; Plague; Primary Care; Psychosocial; Rift Valley; SARS; Sector-wide approach; Service delivery; Smallpox; Social Determinant; Supply system; Surveillance; SWAP; System strengthen-; System strengthening; System-Wide; Tularemia; UHC; Universal Access; Vaccine; Vector control; Virus.

The key term search for investments outside of the health sector identified 4,268 aid activities, with a funding volume of US$4.3 billion. We assessed the largest 1,135 aid activities based on the ‘USD Disbursement’ column (US$3.9 billion), equivalent to 90% of the total funding. Most of the aid activities identified through our search included components were not health related. Therefore, a different framework than that used for classifying the health sector was necessary.

The aid activities identified through the key term search were classified as either health security or system-wide HSS. We used a semi-automated approach to assess these projects. Based on the information in the CRS, the projects were first classified on a numeric system: ‘0’: There is no explicit reference to health or direct benefit to an area relevant to health; ‘1’: Health is only one of many elements explicitly stated in CRS long description. Health accounts for approximately 25% of the project’s resources; ‘2’: Health-relevant components account for approximately half of an activity’s components. Health accounts for approximately 50% of the project’s resources; ‘3’: Nearly all elements explicitly stated in CRS long description are directly relevant to health. Health accounts for approximately 75% of the project’s resources; ‘4’: All elements explicitly stated in CRS long description are directly relevant to health. Such a scaling method was used by Grépin *et al.* in their assessment of donor funding for health policy and systems approach ^27^. Appendix, Panel 2, *Extended data* provides examples of our classification method ^19^.

**Figure 2.**
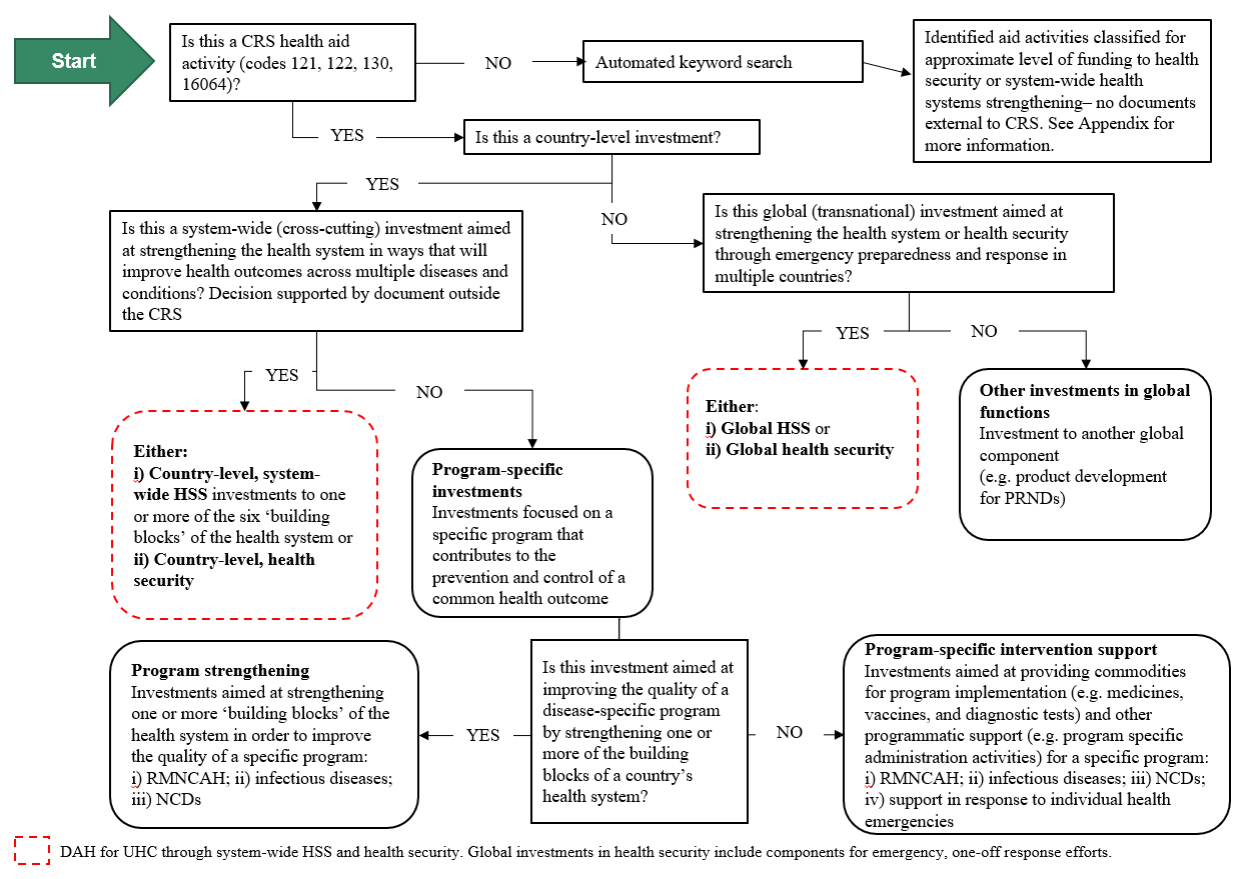
Decision tree that guided the coding of DAH. DAH, development assistance for health; CRS, Creditor Reporting System; HSS, health systems strengthening; PRNDs, poverty-related and neglected diseases; RMNCAH, reproductive, maternal, newborn child and adolescent health; NCD, non-communicable diseases.

The decision tree ( Figure 2) summarizes how the classification process was operationalized.

Finally, we analyzed the overall fit between our estimates of DAH flows and country needs through a cross-sectional analysis, fitting ordinary least squares (OLS) regression models that estimate the relationship between flows of DAH to system-wide HSS and health security and measures of country capacity. Analyses were run in Stata 14.2. We used maternal mortality ratio (MMR; modeled estimates of maternal mortality per 100,000 live births) as a proxy for general health system capacity, and joint external evaluation (JEE) core capacity scores as a measure of national preparedness to prevent, detect, and respond to health security threats.

We controlled for gross domestic product (GDP) and population and estimated three main models to assess the relationship between DAH for health system strengthening and health system capacity, the latter proxied by estimated MMR. Model 1 was a bivariate regression, model 2 included GDP as a covariate, and model 3 included population as a covariate. GDP and population are correlated, and we did not include both variables in a single regression to avoid introducing multicollinearity. MMR is positively and significantly associated with DAH in all specifications. We conducted additional robustness checks, with alternative model specifications including log-transformed independent variables, as well as alternative measurements of maternal mortality (including lagged models and averaging maternal mortality rates over the period 2010–2014), with consistent results.

We estimated three main models to assess the relationship between DAH health security and health security capacity, measured by JEE core capacity scores. As above, model 1 was a bivariate regression, model 2 included GDP as a covariate, and model 3 included population. We expected that lower JEE scores would be associated with higher DAH health security flows (or conversely, that better-prepared countries would receive lower DAH flows). To check the robustness of our results, we estimated additional models using log-transformed GDP and population variables, dropped extreme outliers, and included a binary variable for three primary countries impacted by the 2014–2015 West Africa Ebola outbreak, which received large inflows of DAH health in 2015, with no substantive changes in results. All models omit two high-leverage outliers (countries with DAH health security exceeding 40,000,000 USD, and with an average (rescaled) JEE measure of 80), but results were robust to their inclusion once control variables were introduced.

Data on 2015 country GDP, population, and MMR were extracted from the World Bank World Development Indicators (see *Source data)*. JEE scores were downloaded from Resolve to Save Lives, which abstracted JEE scores from published mission reports; we estimated an aggregate JEE score by taking the mean value of all JEE indicators (results reported here use a rescaled version of the variable, ranging from 0–100). At the time of analysis, 88 countries had completed the JEE process and published mission reports, of which 71 were aid recipients represented in the DAH dataset.

## Results

### Overall levels of DAH for UHC through system-wide HSS and system-wide health security

**Figure 3.**
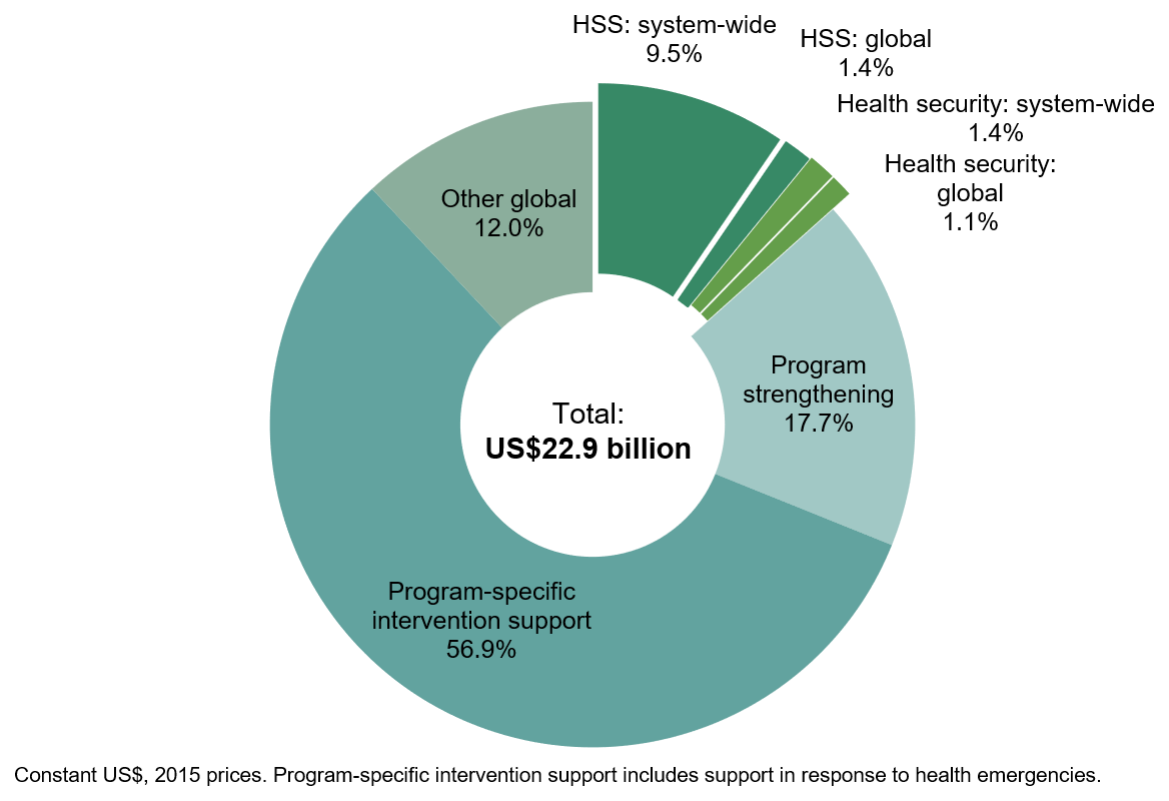
Distribution of total development assistance for health in 2015. HSS, health systems strengthening.

In 2015, of the US$22.9 billion in DAH disbursements in the CRS, we estimated that US$3.1 billion (13.4% of total DAH) was for UHC through system-wide HSS (US$2.5 billion, 10.9% of total DAH) and system-wide health security (US$570.0 million, 2.5% of total DAH) ( Figure 3). The remaining US$19.8 billion of total DAH was for program-specific intervention support (56.9% of total DAH), program strengthening (17.7% of total DAH), and other global investments (12.0% of total DAH).

**Figure 4.**
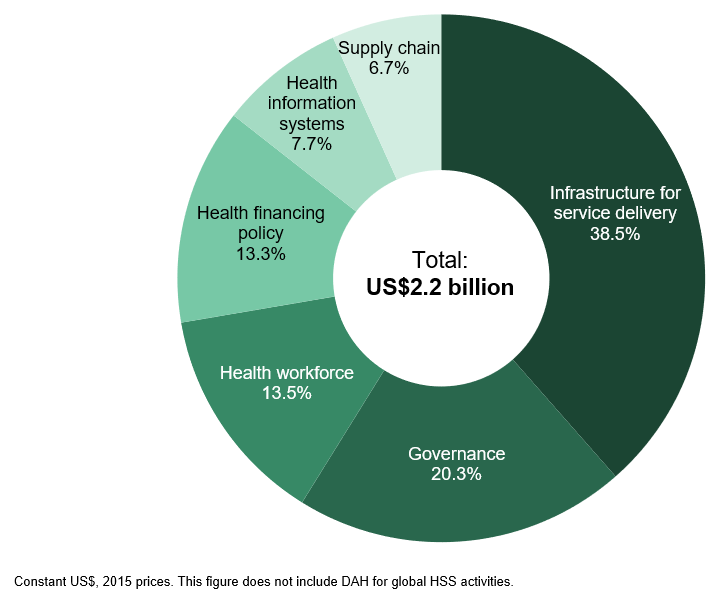
Distribution of system-wide health systems strengthening funding across major health system components. DAH, development assistance for health.

**Figure 5.**
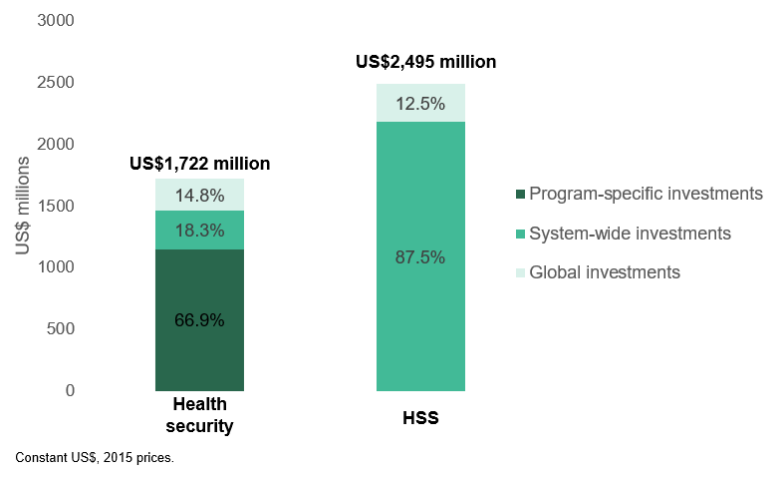
Distribution of development assistance for health for health security and health systems strengthening (HSS) across three major components.

Of the US$2.5 billion in system-wide HSS support, US$2.2 billion was country-level DAH (9.5% of all DAH; Figure 3), and US$311.4 million (1.4% of all DAH) was ‘global’. Infrastructure, governance, and workforce together accounted for about 72% of all country-level DAH for HSS; the remaining 28% was for the strengthening of countries’ health financing policies, health information systems, and supply chains ( Figure 4). In addition, we identified approximately US$417.3 million towards system-wide HSS in purpose codes outside of our definition of DAH, largely for infrastructure and water supply ( Figure 5). This brings our total estimate of donor support for HSS to US$2.9 billion.

A total of US$1.7 billion in DAH was disbursed by donors for health security (7.5% of total DAH) ( Figure 5). About two-thirds (66.9%) of this funding was program-specific investments in response to individual health emergencies (not counted towards our estimate of funding for UHC through health security), and 18.3% was for more fundamental system-wide support (included in our estimate of UHC through health security). Very little financing was found for health security at the global level (US$255.7 million), equivalent to 14.8% of all DAH for health security, or just 1.1% of total DAH. We identified an estimated US$1.9 billion in additional funding for health security through purpose codes outside of our definition of DAH, largely as humanitarian aid in response to emergencies. This brings the total amount of donor support for all health security to US$3.6 billion.

### Distribution of DAH for system-wide HSS, and of DAH for all health security, by recipient income group and region

Of the total DAH invested in 2015, low-income countries (LICs) received 31.2%, lower-middle-income countries (LMICs) received 31.3%, and upper-middle-income countries (UMICs) received 5%. The remainder was regional, unspecified investments, or for global purposes.

- **System-wide HSS.** LICs and LMICs accounted for 69.3% of all DAH for system-wide HSS in 2015 (more details are in Appendix, Table 7–8, *Extended data*) ^19^. In terms of regional distribution, the region that received the largest share of DAH for system-wide HSS was the Africa Region (38.5% of DAH for system-wide HSS at the country or regional level), followed by the Western Pacific Region (13.6%) and the South-East Asia Region (12.4%). The remainder was for the three other WHO regions (Eastern Mediterranean, the Americas, and Europe) and for global support.
- **Health security.** Of the total disbursements for health security (US$1.7 billion), LICs accounted for 30.8% (US$530.2 million), while LMICs received 22.6% (US$388.6 million) and UMICs 4.5% (US$78.3 million). The remainder was regional, unspecified investments, or for global purposes. Nearly a quarter (23.7%) of the total DAH for health security was allocated to the three countries that were most heavily affected by the 2014-2015 Ebola outbreak in Western Africa – Liberia, Sierra Leone, and Guinea. The countries with the highest ratio of health security support to total DAH were the West Bank and Gaza Strip, Yemen, and Liberia, all on the OECD’s 2015 list of fragile states (Appendix, Table 9–10, *Extended data*) ^19^.

### Distribution by donor

The proportion of DAH provided by donors to these areas varied widely by donor. Government donors that prioritized system-wide HSS in their DAH portfolios in 2015 were Australia (43.4% of its DAH portfolio went to system-wide HSS), Korea (42.7%), the European Union (38.1%), Germany (36.3%), and Japan (34.0%). Government donors that prioritized health security (including program-specific investments) in their DAH portfolios in 2015 were France (17.4% of its DAH portfolio went to health security), Japan (13.1%), and Germany (13.0%). The International Development Association (IDA) and WHO contributed over half of all multilateral support for system-wide HSS (41.3% and 21.2%). IDA and the United Nations Relief and Works Agency for Palestine Refugees in the Near East contributed over half of all multilateral support for health security (33.4% and 21.7%, respectively).

### Alignment with country need

We found a positive, statistically significant association between DAH flows to system-wide HSS and maternal mortality ratios, suggesting that weaker health systems receive higher levels of DAH for HSS. However, we found no statistically significant relationship between JEE scores and DAH for health security (including program-specific support in response to individual health emergencies).

Across all model specifications, we did not find a correlation between DAH for health security and country capacity to respond to public health risks, as measured by the JEE, a finding that may reflect data limitations - several JEE evaluations are in progress or yet to be completed (Appendix, Panel 3, *Extended data*) ^19^. This is an important avenue for future research, as LICs and MICs with the weakest health systems and greatest vulnerability to health emergencies are likely to also have greater difficulty mobilizing domestic financing for these functions and a greater need for DAH. Appendix, Panel 3, *Extended data* provides more detailed findings on the regression analysis findings ^19^.

## Discussion

In 2015, we estimated that donors invested US$2.2 billion in DAH for system-wide health security. In addition, we estimated US$1.9 billion for health security outside of the health sector, including response efforts and US$417 million for system-wide HSS.

Our estimate of overall donor spending for system-wide HSS is similar as a proportion to the estimate by the Institute for Health Metrics and Evaluation (IHME). Although IHME used a very different methodological approach and covers additional financing (Appendix pp. 20–21, *Extended data*) ^19^, it found that 9.4% of total DAH went towards HSS in 2015, nearly identical to our study’s estimate of 9.5% for system-wide HSS (both estimates exclude investments that are transnational). IHME does not produce detailed project-level data, thus our disaggregated estimates for HSS by cannot be compared by building block. IHME reports that only 0.8% of DAH in 2015 was directed at pandemic preparedness, slightly below our most comparable estimate (1.4% of total DAH for system-wide health security, i.e., excluding programmatic support in response to health crises and global investments) ^23^. We found significantly less financing for the health workforce than Micah *et al.* found ^13^, which may reflect differences in methodologies.

The study illuminates three important findings on how DAH is channeled and directed. First, we found that most investments in health system was reactive to emergencies, rather than proactive investments in preparedness (such as pandemic preparedness). Thus, it is likely that donors are massively under-investing in preparedness relative to need ^11^. Second, our study found that 12.5% of all DAH for HSS and 14.8% of DAH for health security in 2015 was delivered at the global level, compared to 14.5% of overall DAH (a total of US$3.3 billion). This suggests that DAH for HSS relative to health as a whole is more focused on benefiting specific, individual countries. A key question going forward is how much donors *should* be spending on global functions versus country-specific support? Yamey and colleagues argue that donors will see higher returns if they rebalance their portfolio towards the global level ^28^. Thirdly, we found that LICs (the countries with the least ability to fund health domestically) accounted for 31% of all DAH for system-wide HSS in 2015. Going forward, donors should consider targeting LICs when they make their resource allocation decisions as DAH is likely to play an important role in funding HSS in LICs. Collectively, these findings can be used to help guide evidence-based decision-making for global health donors who are looking to understand how much DAH is available for the reduction of cross-border threats and the strengthening of health systems. They also provide important data for monitoring and advocacy related to closing the financing gap for achieving UHC at large.

We believe our study is particularly timely. Experts believe that the devastating health and economic consequences of the novel coronavirus disease 2019 (COVID-19) serve as a reminder of the vulnerability transnational health threats pose on health systems and the importance of the SDGs, especially UHC, in curbing pandemics and other transnational health threats ^29^. Additional investments in country health systems and the global health system in particular will be needed to more effectively prevent and curb transnational health threats. Going forward, it may be appropriate for national governments to thus lead the response to investment needs in HSS and for donors to lead the response on global functions and health security ^28^. Monitoring support for these areas serves as a way for holding donors accountable for these investments, informing evidence-based policy decisions and measuring progress going forward. Our study creates a framework for monitoring trends that is well-aligned with Stenberg and colleagues’ framework for assessing financing needs. Our framework may thus create a structure and methodology for more closely monitoring the investment gap going forward ^8^.

There are at least seven key limitations to our study. First, our assessment focused on 2015 investments alone, so we are unable to make any inferences about time trends. Second, our analysis is limited to disbursements by donors who report their investments to the CRS. It includes US$13.2 billion less in DAH financing for 2015 than is captured by IHME. As such, it does not capture corporate donations, DAH from China, and some multilateral funding that is captured by IHME using additional data sources. Our estimates should thus be interpreted as underestimates. Third, our study focuses on disbursements rather than commitments, meaning that aid activities may not reflect the intent of the donors. That said, disbursements are closer to funds available. Our database contains projects without disbursement data at the time of the analysis (approximately US$140 million in additional financing). This reflects that our study relies on a CRS database close to the end of the reporting year. It may also refer to projects for which a donor is reporting a commitment but has not yet disbursed. Future studies could avoid this by predicting disbursements from commitment data. Forth, we included a purpose code outside of the OECD’s definition of aid to health (‘social mitigation of HIV’) in our estimate of DAH. Given that we have not included other related purpose codes in our definition of DAH, this creates a slight bias. Fifth, we based the assessment of aid activities outside of the health sector based only on the limited information available in the CRS. Sixth, our analysis relies on a review by multiple analysts of limited available information against a highly complex framework. Although we put efforts in place to harmonize the classification, our method is vulnerable to imperfect inter-coder reliability. Finally, although the approach of using a non-random sampling technique was taken to capture the greatest amount of financing with available resources, such an approach introduces a sampling bias and means we have not captured the contents of smaller projects.

A future study should be expanded to include domestic financing, to cover multiple years, to more critically assess support outside of the health sector, include commitments in addition to disbursements, and to assess donors that do not report to the CRS. Improvements could also be made to the CRS to better deliver on this method and to measure support for UHC at large – for example, by including more donors, encouraging more descriptive information, or by introducing a ‘policy marker’ for UHC. A policy marker is a way of tagging or marking aid activities that target a particular focus area ^30^. This study reconfirms that tracking techniques need to take into account trade-offs between various factors like timeliness, transparency, precision, and replicability ^31^. It also reinforces the need for the global health community to work towards a common definition of how DAH is defined going forward and to consider how country need is taken into account when making financing decisions, particularly regarding health security investments. We suggest that a future study explores the relationship between country need and financing in more detail (e.g., by examining system-wide health security and other estimates of pandemic preparedness against JEE since the JEE is mainly about managing transboundary risks and not localized humanitarian crises). We hope that our analytical framework can be leveraged for identifying additional health investments needed to achieve UHC and SDG3.

## Data availability

### Source data

The original source file for the underlying aid activity data is the OECD DAC’s CRS dataset, available on the OECD’s website: http://stats.oecd.org/Index.aspx?datasetcode=CRS1. The dataset for this analysis is based on the OECD’s June 2017 update of the 2015 CRS database. The file can be found by clicking ‘Export’, ‘Related files’, then by selecting the “CRS 2015 data.zip” file. The file was the most up to date 2015 data file at the start of the analysis.

For the regression, data on 2015 country GDP, population, and maternal mortality ratios were extracted from the World Bank World Development Indicators ( https://data.worldbank.org/indicator/NY.GDP.MKTP.CD; https://data.worldbank.org/indicator/SP.POP.TOTL; https://data.worldbank.org/indicator/SH.STA.MMRT, respectively). The JEE scores were abstracted and structured by Prevent Epidemics, and were extracted in August 2019 ( https://preventepidemics.org/wp-content/uploads/excel/all-countries.xlsx).

### Underlying data

Open Science Framework: Development assistance for UHC through health systems strengthening and health security. https://doi.org/10.17605/OSF.IO/B5YAX ^19^.

This project contains the following underlying data:

- Dataset.xlsx

### Extended data

Open Science Framework: Development assistance for UHC through health systems strengthening and health security. https://doi.org/10.17605/OSF.IO/B5YAX ^19^.

This project contains the following extended data:

- Appendix.pdf

Data are available under the terms of the Creative Commons Zero "No rights reserved" data waiver (CC0 1.0 Public domain dedication).

## Code availability

Reproducible code is available at: https://github.com/jessckraus/dahuhc/tree/v1.0.

Archived code at time of publication: https://doi.org/10.5281/zenodo.3858071 ^26^.

License: Creative Commons Zero "No rights reserved" data waiver (CC0 1.0 Public domain dedication).
