# Supplementary material for: Measuring development assistance for health systems strengthening and health security: an analysis using the Creditor Reporting System database
Source: F1000Res. 2020 Jun 9;9:584. [Version 1] doi: 10.12688/f1000research.24012.1 (PMC9156894; doi:10.12688/f1000research.24012.1)
Supplement: Peer review history

# Peer review: Reviewer response for version 1

**DOI:** 10.5256/f1000research.26488.r94393

**Published:** 2022-05-31

**Authors:** Ashley Fox, Sana Abdelkarim

**Affiliations:** 1 Rockefeller College of Public Affairs and Policy, University at Albany, State University of New York, Albany, New York, USA; 2 Rockefeller College of Public Affairs and Policy, Albany, NY, USA

The article by Kraus *et al.* offers an important analysis of how much development assistance for health is allocated towards specific activities of health system strengthening (HSS) and health security which are important pillars to achieving universal health coverage. The article provides a great step towards attempting to count development assistance for health (DAH) towards specific health issues and disaggregating the flow by the building blocks of the health system. Furthermore, the study marks an essential step towards expanding our understanding of not only the impact of DAH on HSS and health security but also towards our ability to better comprehend the contribution of each of the building blocks to achieving Sustainable Development Goals including universal health coverage.

 The study approach coveys the authors' understanding of the data and sources. The study design seems appropriate for tracking DAH flow using Creditor Reporting System (CRS). The authors provide a clear description of data sources and a careful, detailed explanation of the methodology which allows for replication. Although the analysis is pretty complex, the authors presented an excellent and meticulous illustration of their process and provided a useful framework to understand the DAH flow towards HSS and health security.

 The authors’ classification of DAH flow included country-region-specific health programs, system-wide programs, and funds contributing to global function which provide a good view of DAH health investment. When applying the framework to CRS, the authors attempted to provide an accurate calculation of aid activities. However, their attempt to accurately calculate DAH flow was met with an inability to follow the same process to count the contribution of other sectors to HSS and health security. The authors then had to conduct additional CRS purpose codes using key search terms which was a necessary step to achieve a higher level of accuracy but a complex step because the search terms revealed aid activities that are not related to health. The authors then had to swift through these outputs and further code based on the estimation of health-relevant components of the allocations. Nevertheless, the authors described, in good detail, all decisions that needed to be made and their rationale which shows the high level of transparency accompanying the work.

 The authors analyze the data using linear regression models to measure the relationship between DAH flow and the country’s health system capacity using maternal mortality rates (MMR) as a proxy controlling for GDP and population and found a strong positive association between DAH flow and MMR. The authors also used a similar model to estimate the relationship between DAH flow and the country’s need using joint external evaluation (JEE) as a measure for health security capacity and found no significant relationship between them which they credited to data limitation from JEE score. I think this might be the weakest point of the study because of the data limitation from JEE (limitation of MMR as a proxy for health care capacity). In addition to the limited ability of linear regression to provide strong associations of country-wide measures without controlling for countries' specific effects beyond GDP and population.

 In the discussion section, the authors provided an important comparison between their estimate and the IHME estimates of DAH flow and found a close resemblance which provides further confidence in the results.

 This study is very important and timely due to the expected economic downfalls resulting from the COVID-19 pandemic. The conclusion highlights the important contribution this study provides. The framework allows an opportunity to expand our understanding of the impact of DAH on HSS and health security. The study highlights the importance of creating more investments by allocating funds to strengthen the health care system and improve pandemic preparedness, especially for low- and middle-income countries.

**Comments/questions:**

**Introduction **

- The third paragraph contains the first reference to the building blocks of the health system and then later in the methodology they reference the six building blocks of the health system presented in Stenberg *et al*.’s 2017 conceptual framework. Could you please clarify the building blocks framework at the beginning? Stenberg *et al*.’s 2017 conceptual framework include similar to WHO building blocks, which I believe are introduced in 2010 here. The only difference I noticed is in the terminology of “medical technology: in the WHO and “Supply chain” in Stenberg *et al.*’s framework.

**Methods**

- Page 4: Can you clarify the rationale behind including "…a component for ‘system-wide emergency preparedness, risk management and response’ for activities that strengthened capacity to deal with epidemic and pandemic preparedness and response, e.g., a mobile emergency alert system" with the system-wide HSS and not with “Global functions”, given that pandemic and endemic preparedness have as much of a transnational benefit as a local/domestic effect.
- Page 5: “…to arrive at a high-level estimate of total donor support for HSS and health security, we expanded our analysis and conducted **a light-touch analysis** of additional CRS purpose codes). We conducted a key term search using Python 3.6 across 14 additional CRS purpose codes” - Can you clarify what is “light-touch analysis”?

Is the work clearly and accurately presented and does it cite the current literature?

Yes

If applicable, is the statistical analysis and its interpretation appropriate?

Yes

Are all the source data underlying the results available to ensure full reproducibility?

Yes

Is the study design appropriate and is the work technically sound?

Yes

Are the conclusions drawn adequately supported by the results?

Yes

Are sufficient details of methods and analysis provided to allow replication by others?

Yes

Reviewer Expertise:

Global health policy analysis; health politics and policy; political economy of development.

We confirm that we have read this submission and believe that we have an appropriate level of expertise to confirm that it is of an acceptable scientific standard.

# Peer review: Reviewer response for version 1

**DOI:** 10.5256/f1000research.26488.r83868

**Published:** 2021-05-17

**Authors:** Leonardo Arregocés

**Affiliations:** 1 London School of Hygiene & Tropical Medicine, London School of Hygiene & Tropical Medicine, London, UK; 2 Ministry of Health of Colombia, Bogotá, Colombia

This paper from Jessica Kraus, Gavin Yamey, Marco Schäferhoff *et al.* presents an evolution of methods for estimating development assistance for a specific focus area. It presents an analytic framework that allows to classify the transactions reported to the Creditor Reporting System (CRS) into neatly separated investment areas. The framework was then applied to the 2015 data set from the OECD CRS to produce estimates of total and disaggregated disbursements for Development Assistance for Health (DAH). A latter analysis of targeting of DAH disbursements related to need was assessed through regression analysis.

 This paper can be considered another step towards having better tools to analyse development assistance. Although is not a completely new approach, it does make an important improvement in defining a neat framework to classify DAH. The text does go through other approaches to estimate DAH and presents how it compares to those, including the benefits of this new approach.

 The study design shows the knowledge and experience that the authors have on the context and the data sources. The approach decided, which privileges the careful reading of the data in the CRS and further search of missing data in other sources, over including all the records in the dataset that seems appropriate. The process is labour intensive, but the additional effort of searching for additional data was very important. Only one-fourth of the records seem to be based solely on CRS data. The remaining required additional efforts to search for details. That means that not only the effort in classifying was meaningful, but the apportioning of the disbursement into the different categories is quite valuable. Previous similar efforts which included coding all records did not include searching for additional data, relying only on assumptions on the reported information. Although assumptions were adequate, they could not allow for deeper analyses. Technically the work is adequate.

 The methods have been well described. They are clear despite the complexity of the work done. Process of analysis is well described in the text and in the additional data. The data necessary to replicate the work is available, although quite a bit of the work is analyst dependant. The paper describes all measures taken by the authors to ensure consistency in the coding as much as possible. It could be useful to make some quantitative measure of intercoder consistency. The data file annex to the paper makes much clearer how the coding was made and allow to reproduce the results.

 Probably the weakest part of the method are the searches in purpose codes other that health. It is not described the criteria for creating the list of keywords. The purpose of running searches in other purpose codes is to not leave outside the estimates transactions that can count towards health. Assurance that nothing was left out can be shown by explaining the comprehensiveness of the keywords and the proportion of identified records that were included in the estimates. Authors could reflect on possible ways to identify transactions that can be related to health, but are not included in health purpose codes.

 Assessment of targeting of DAH to needs was done using linear regression. This approach has been used elsewhere. Three models were defined using different covariates. In one GDP was used and in the other population size. Is argued that both could not be used at the same time because of collinearity, but probably using GDP per capita would have been a good alternative to adjust for both. The results for the assessment of targeting to need are according to what is expected. Countries with higher maternal mortality rates receive more support. Coefficients for GDP is also positive suggesting that countries with higher incomes receive more money. This is not discussed in the paper and it seems counter intuitive. This might be suggesting that the relation between DAH and need is not linear, although authors used a log – log specification and found the same results. Probably donors prefer to target the more developed countries. A scatter plot of DAH and GDP labelling countries with different colours according to a categorised level of income.

 Data sources are well described, and a processed data set is made available. Besides the paper, producing a dataset that can be further used for other types of analysis is a valuable contribution. Many questions can be answered with the available data. A problem with the dataset for further use is that some columns from the CRS are missing. If the unique ID were generated by the authors, I would suggest making available the same dataset with all columns in the CRS for the selected records so further analyses are feasible.

 Discussion and conclusion reflect on the main points. All the topics covered in the paper are touched, although they are very broad. Being this a paper that is presenting an improvement on previous methods, which has a big potential for deeper analyses of the effectiveness of DAH in achieving SDGs, I would suggest reflecting more on the precision of the estimates, the effort and plausibility of continuing this work, and probable future improvements. Because this kind of approach is labour intensive, some discussion on the means to keep processing the data could be valuable so this is not a one time only. This can be accompanied by the future uses the authors see of the estimates and further questions that can be answered.

 This paper covers many topics which makes it quite complicated to stuff all that in a few thousand words. Authors do a great job in that aspect, leaving behind other reflections that are necessary. The way the method is describes and the data that accompanies the paper is good proof of a very hard work and high quality work.

Is the work clearly and accurately presented and does it cite the current literature?

Yes

If applicable, is the statistical analysis and its interpretation appropriate?

Partly

Are all the source data underlying the results available to ensure full reproducibility?

Yes

Is the study design appropriate and is the work technically sound?

Yes

Are the conclusions drawn adequately supported by the results?

Yes

Are sufficient details of methods and analysis provided to allow replication by others?

Yes

Reviewer Expertise:

I have previously worked on estiamting development assistance to reproductive, manternal, newborn, and child health using a similar method to the one used by the authors. Also participated in publications that reflec on methods for estimating disbursements of develpoment assistance to health. I know the CRS data quite well.

I confirm that I have read this submission and believe that I have an appropriate level of expertise to confirm that it is of an acceptable scientific standard.
